# Digital Quantification of Gene Expression in Sequential Breast Cancer Biopsies Reveals Activation of an Immune Response

**DOI:** 10.1371/journal.pone.0064225

**Published:** 2013-05-31

**Authors:** Rinath M. Jeselsohn, Lillian Werner, Meredith M. Regan, Aquila Fatima, Lauren Gilmore, Laura C. Collins, Andrew H. Beck, Shannon T. Bailey, Housheng Hansen He, Gilles Buchwalter, Myles Brown, J. Dirk Iglehart, Andrea Richardson, Steven E. Come

**Affiliations:** 1 Center for Functional Cancer Epigenetics, Dana-Farber Cancer Institute, Boston, Massachusetts, United States of America; 2 Department of Medical Oncology, Dana-Farber Cancer Institute and Department of Medicine, Brigham and Women's Hospital, Harvard Medical School, Boston, Massachusetts, United States of America; 3 Department of Biostatistics and Computational Biology, Dana-Farber Cancer Institute and Department of Medicine, Harvard Medical School, Boston, Massachusetts, United States of America; 4 Department of Cancer Biology, Dana Farber Cancer Institute, Boston, Massachusetts, United States of America; 5 Department of Pathology, Beth Israel Deaconess Medical Center, Harvard Medical School, Boston, Massachusetts, United States of America; 6 Department of Surgery, Brigham and Women's Hospital, Harvard Medical School, Boston, Massachusetts, United States of America; 7 Department of Pathology, Brigham and Women's Hospital, Harvard Medical School, Boston, Massachusetts, United States of America; 8 Breast Medical Oncology Program, Beth Israel Deaconess Medical Center, Harvard Medical School, Boston, Massachusetts, United States of America; Karolinska Institutet, Sweden

## Abstract

Advancements in molecular biology have unveiled multiple breast cancer promoting pathways and potential therapeutic targets. Large randomized clinical trials remain the ultimate means of validating therapeutic efficacy, but they require large cohorts of patients and are lengthy and costly. A useful approach is to conduct a window of opportunity study in which patients are exposed to a drug pre-surgically during the interval between the core needle biopsy and the definitive surgery. These are non-therapeutic studies and the end point is not clinical or pathological response but rather evaluation of molecular changes in the tumor specimens that can predict response. However, since the end points of the non-therapeutic studies are biologic, it is critical to first define the biologic changes that occur in the absence of treatment. In this study, we compared the molecular profiles of breast cancer tumors at the time of the diagnostic biopsy versus the definitive surgery in the absence of any intervention using the Nanostring nCounter platform. We found that while the majority of the transcripts did not vary between the two biopsies, there was evidence of activation of immune related genes in response to the first biopsy and further investigations of the immune changes after a biopsy in early breast cancer seem warranted.

## Introduction

Window of opportunity clinical trials or non-therapeutic studies in breast cancer are studies that exploit the time interval between the diagnostic core needle biopsy (CNB) and definitive surgery for a brief exposure to a study drug. As opposed to neoadjuvant studies, in which patients are given an investigational therapy for a prolonged period of time and the end point is usually clinical or pathological response, the intervention in window of opportunity studies is brief and the goal is not therapeutic but rather the biological changes that occur in response to the intervention. This model of clinical investigation cannot replace the gold standard large randomized adjuvant trials, which provide important data about long-term exposure to a given agent, particularly data about toxic effects. However, in an era where only a tenth of new targeted agents that enter clinical development are approved by the US Food and Drug Administration [1], window of opportunity studies can serve as a relatively rapid and low-cost tool to exclude ineffective agents from further development and validate markers that can define a subset of patients that benefit most from a new agent or novel combinations of agents. Moreover, this approach allows access to tumor tissues before and after treatment in close to 100% of enrolled patients.

Over 75% of breast cancers are estrogen receptor positive (ER+) and despite effective endocrine treatments, most breast cancer-related deaths occur within this sub-group of patients [2]. This underscores the need for new-targeted agents combined with hormonal blockade to improve outcomes in patients with early ER+ breast cancer. The investigation of new agents for early ER+ breast cancer requires prolonged follow-up and very large sample sizes because of the low annual risk of recurrence but protracted risk over several decades. Thus, window of opportunity studies have the potential to be an important modality for the study of hormonal blockade combined with new agents targeting ER+ breast cancer. The IMPACT (Immediate Preoperative Anastrazole, Tamoxifen, or combined with Tamoxifen) trial is one of the first studies to show early molecular changes in ER+ breast cancer after a brief exposure to hormonal treatment. In this study, 330 post-menopausal women were randomized to 12 weeks of pre-operative anastrazole, tamoxifen or anastrazole combined with tamoxifen. Ki67 was selected as the primary biomarker end-point and changes in Ki67 levels were detected after just two weeks of treatment. The level of Ki67 suppression differed between the anastrazole and tamoxifen arms and mirrored the recurrence free survival results of the ATAC (Arimidex, Tamoxifen Alone or in Combination) adjuvant trial [3], [4]. Furthermore, in a later analysis, Ki67 levels at a two week point correlated with recurrence free survival suggesting that the early changes in Ki67 after a brief exposure to endocrine treatment is indicative of long term outcomes [5].

Other window of opportunity studies using novel agents in ER+ breast cancer have been published recently [6]–[9]. One such study investigated a short exposure to presurgical erlotinib in newly diagnosed breast cancers and showed that in ER+ breast cancers and not HER2+ or triple negative disease, there was a reduction in the phosphorylation of both ER at the S118 site and of MAPK as well as a Ki67 response that was not dependent on EGFR positivity. This study suggests that erlotinib is worthy of further investigation in ER+ breast cancers and not Her2 positive or triple negative breast cancers and EGFR positivity does not necessarily predict response [6].

Non-therapeutic studies have several drawbacks; while the Ki67 marker has been extensively studied and incorporated into such studies and is a also a key component of Oncotype Dx and other multi-gene expression prognostic test [10], [11], it is not a useful biomarker of response for all types of targeted treatments, e.g. agents that target angiogenesis or apoptosis. Moreover, in most studies Ki67 is quantified by immunohistochemistry, which is an assay that is prone to both technical errors and interpretation bias. Hence, additional biomarkers that can predict treatment responses and long-term outcomes as well as assays that are highly reproducible and quantitative are needed. In addition, since the end point of window of opportunity studies is molecular changes it is critical to define gene expression variations between core biopsies and excisional biopsies in the absence of drug exposure. Such variations may occur due to smaller sampling size in the core biopsies and tumor heterogeneity, differences in tissue handling and processing or as a result of the core biopsy itself. The aims of this study were to determine if there are variations in gene expression levels between core biopsies of early breast cancer specimens compared to the matched excisional biopsies (EB) in the absence of any intervention using a highly reproducible and quantitative assay, the Nanostring nCounter system, and study the correlation between clinical immunohistochemistry protein expression levels and the Nanostring transcript expression level [12], [13]

## Materials and Methods

### Ethics statement

The prospective collection of breast cancer tissue samples from core biopsies and excisional biopsies was done with patient consent and the Dana Farber/Harvard Cancer Center Institutional Review Board approval under protocol #07-104.

### Patient samples

Tissue was collected from post-menopausal women with newly diagnosed breast cancer with a primary tumor larger than 1 cm on the basis of physical exam or imaging. At the time of a diagnostic core biopsy, 2–4 extra core needle biopsies with a 14-gauge needle were obtained and immediately snap frozen in 2-methyl-butane in Optimal Cutting Temperature Compound. Patients who were diagnosed with invasive carcinoma underwent definitive surgical resection and at that time a paired specimen was obtained and snap frozen. Presence of invasive cells was verified by hematoxylin and eosin staining and RNA was isolated using Trizol (Invitrogen) in accordance with the manufacturer's instructions.

### NanoString nCounter analysis

RNA concentration was measured with the Nanodrop 1000 (Nanodrop). Color coded barcodes that represent a single target transcript were synthesized targeting 147 breast cancer related genes and 6 control housekeeping genes (Nanostring technologies). The barcodes were hybridized overnight to 100 ng of total RNA in a reaction that includes a hybridization buffer and a capture probe. The latter enables the immobilization of the complex for data collection. Following incubation, samples were placed on a prep station where excess probes were removed and the probe-transcript complexes were immobilized on a streptavidin coated cartridge. Subsequently the cartridges were placed in the Digital Analyzer and barcodes were counted and tabulated. Each count represents one molecule. Raw nanoString counts were subjected to a technical normalization using positive control spikes that corrects for any experimental variables between the samples (e.g. differences in efficiency in hybridization, purification or binding). This normalization is done by multiplying all counts by a normalization factor that is calculated by the average of the sum of the counts for all positive hybridization controls for each sample divided by the sum for each sample. The normalization factor was between 0.3–3 (within the required range). Data is also normalized for RNA content using housekeeping genes and multiplying all counts by a calculated normalization factor. Because of the potential variability in the expression of housekeeping genes, in order to optimize the normalization, multiple housekeeping genes were used including genes with high and low expression (CLTC, GAPDH, GUSB, HPRT1, PGK1, TUBB). This normalization factor is calculated by the average of the geometric mean of all the housekeeping gene counts for each sample divided by the geometric mean of each sample. The normalization factors ranged between 0.3–7.6 (for valid data the values are required to be within a range of 0.1–10). Nanostring count levels of the housekeeping genes are shown in table S1. Additionally, there are eight codes that have no transcript that are used as negative controls for background noise by subtracting the average of the negative controls from the normalized gene counts.

### Immunohistochemistry

Immunohistochemistry was performed on formalin fixed paraffin embedded sections. The antibodies used were as follows: ER (Neomarkers), PR (Dako), HER2 (Dako), Ki67 (Vector Labs VP-RM04) and CD68 (Dao M0876). Nuclei were counterstained with hematoxylin. The staining was performed by the pathology department at Beth Israel Deaconess Medical Center and the Dana Farber/Harvard Cancer Center research pathology core. All samples also had FISH analysis for HER2 amplification. A HER2/cen17 ratio above 2.2 was considered positive. All samples were reviewed by a pathologist and scoring for ER as follows; zero percent positive cells among all invasive cells was scored as negative, 1–10% low positivity and above 10% is positive.

Scoring of the immunohistochemistry staining of Ki67 was done by counting at least 1000 invasive tumor cells in at least three different high power fields and in accordance with published guidelines [14]. The score was expressed as the percentage of positively staining cells among the total number of invasive tumor cells in the area. Assessment of CD68 was also done by counting at least 1000 invasive tumor cells in three different fields and scoring is the percentage of positive cells among the total number of invasive tumor cells.

### Real-time PCR

Equal amounts of RNA were used as templates for cDNA synthesis using the Applied Biosystems kit and PCR reactions were carried out using an ABI Prism 7700 Sequence Detection System (Applied Biosystems). The fold change expression between the core biopsy and excisional biopsy for each gene was calculated using the ΔΔCt method with GAPDH mRNA as an internal control. The primers used are shown in table S4.

### Statistical Analysis

Differences in protein and mRNA transcript expression levels between the core and excisional biopsies were assessed using Wilcoxon signed rank tests. Spearman correlation coefficients quantified the correlation between measures protein and transcript expression levels. The association of the difference in gene expression between biopsies and the length of the time interval between the biopsies (more than or less than 1 month) was evaluated using Wilcoxon's rank sum tests.

### Clustering

Unsupervised clustering analyses were performed with MeV 4.8 (http://www.tm4.org/mev/) using Pearson correlation with average linkage clustering.

## Results

Sufficient RNA was collected from 23 matched pairs of cores and excisions. Nanostring nCounter gene expression analysis was successful in 21 of the paired biopsies and all the analyses in this study were done on these tumors. All patients were post-menopausal and as expected the majority had ER positive breast cancers. 60% of the patients had stage II disease and grade II disease and 80% of the patients underwent a partial mastectomy as definitive surgery. Further details are shown in table 1.

**Table 1:** Clinical characteristics of the participating patients.

Category	Sub-Category	No. of Patients (%)	Years	Days
** `Age**	Mean		68	
	Range		52–86	
**Interval Between Biopsies**	Mean			30
	Range			6–65
**Definite Surgery**	Lumpectomy	17 (80)		
	Mastectomy	4 (20)		
**Tumor Grade**	I	2 (10)		
	II	12 (60)		
	III	7 (30)		
**Tumor Stage**	I	7 (30)		
	II	11 (60)		
	III	3 (10)		
**Tumor Classification**	Hormone receptor +	18		
	HER2 +	3		
	Triple negative	2		

**Figure 1:**
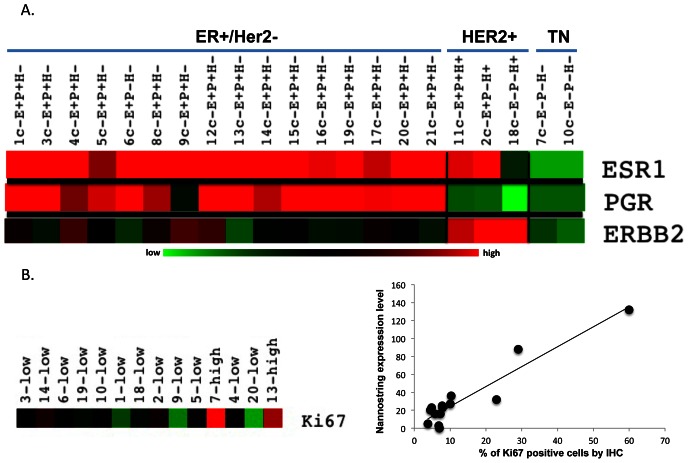
Correlation between CNBs clinical data and Nanostring nCounter expression values. Color coding- green represents low nanostring expression level and red represents high nanostring expression level. Expression levels are log transformed. A. ER+/HER2 negative breast cancers had high ER expression and low HER2 Nanostring expression, HER2 positive tumors displayed high ERBB2 expression levels and triple negative (TN) cancers had low ER and HER2 expression levels. E = ER, P = PR, H = Her2. B. Ki67 high defined as an IHC score of above 14% and low as ≤14%. Scatter plot showing correlation between the Nanostring Ki67 expression level (Y axis) and IHC Ki67 score (x-axis).

Because this study focused on ER+ breast cancers we assembled a list of 147 transcripts to be quantified, which consists of genes that are related to the ER transcriptional network. Included are genes that are ER target genes (e.g. PR, Cyclin D1, XBP1), ER regulators (e.g. AIB1, FOXA1, GATA3), genes that have been implicated in endocrine resistance (e.g. EGFR, HER2, Insulin –like-receptor, PI3K), inflammatory genes and additional related genes [15]–[20]. The list also includes the 16 genes of the Oncotype DX recurrence score and the 50-gene breast cancer intrinsic sub-type classifier (PAM50), since both of these assays have prognostic and predictive properties in ER positive breast cancers [10], [21], [22]. We profiled the RNA extracted from the cores and excisions in the 42 samples using the Nanosting nCounter system. This system is as sensitive as RT-qPCR and does not require the conversion of RNA to cDNA. The full list of genes and normalized data is in table S1. As an internal control we compared the digital transcript counts for ESR1 (encoding ER), PGR (encoding Progesterone Receptor (PR)) and ERBB2 (encoding HER2) to the clinical ER, PR and HER2 status, and these were highly associated (figure 1A). Ki67 score in both the cores and excisions also highly correlated with the Nanostring MKI67 expression (Cores, Spearman r = 0.62, P = 0.02; excisions, Spearman r = 0.60, P = 0.02) (figure 1B). These results validate the integrity of the acquired gene expression data.

**Figure 2:**
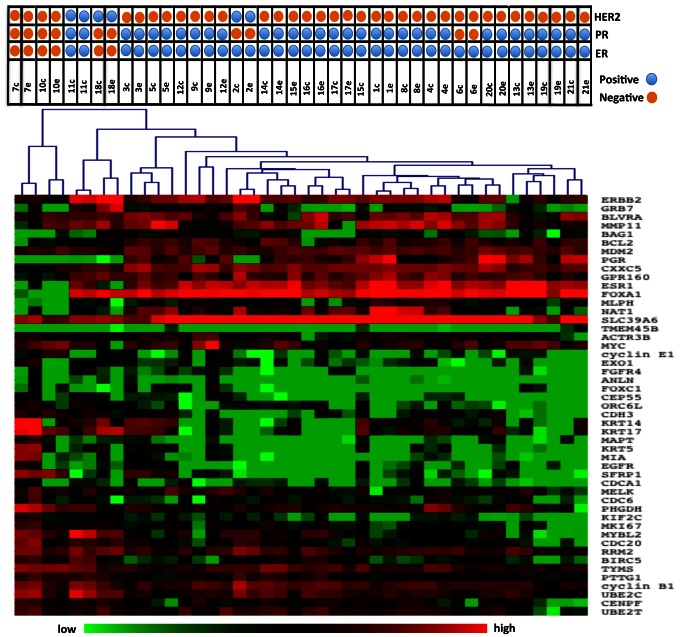
Heatmap of unsupervised clustering of both the CNB and EB samples. Heatmap of unsupervised clustering of all the samples and all the transcripts. All of the CNB and EB pairs co-aggregated at the first level dendrogram.

**Figure 3:**
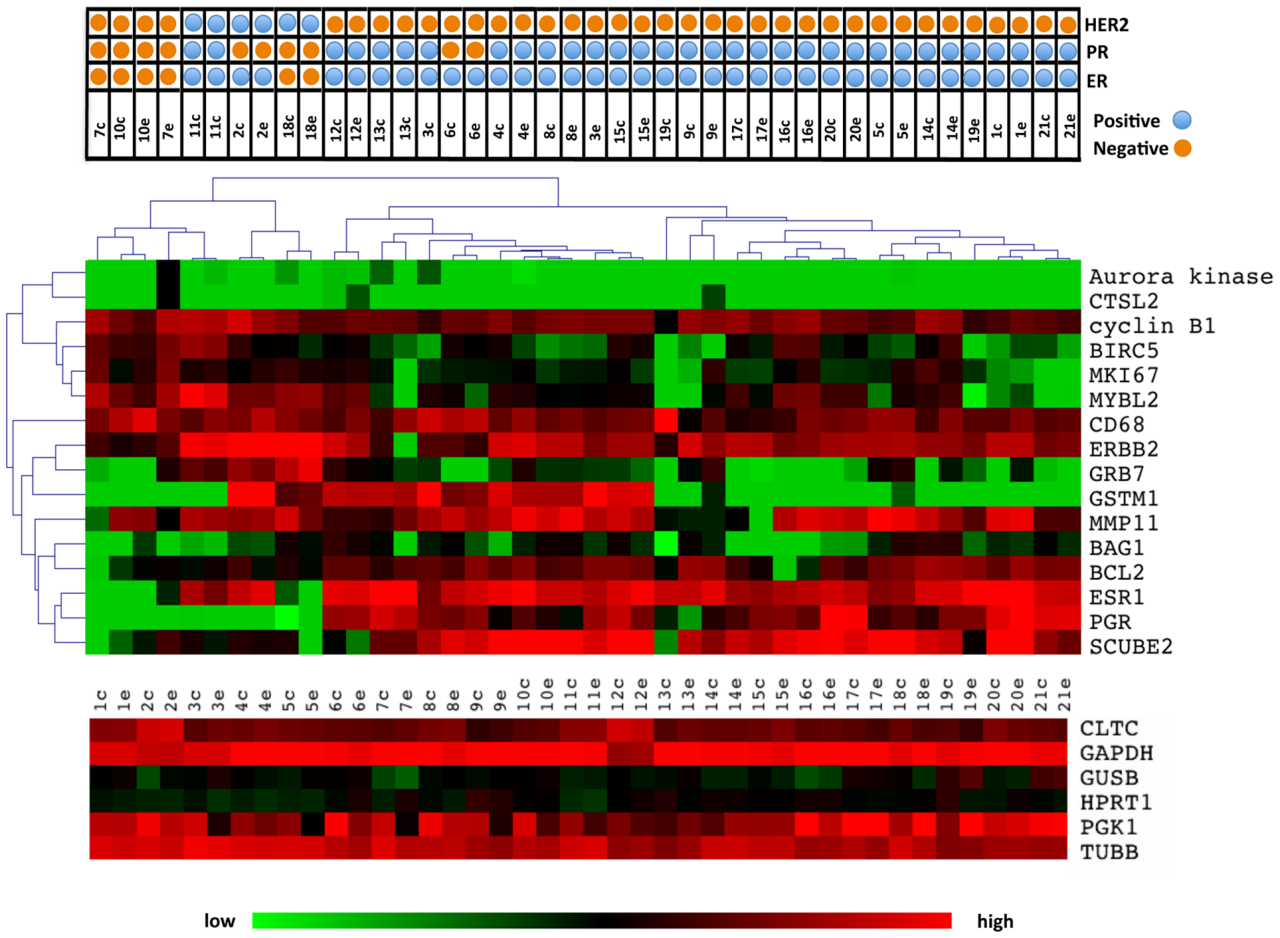
Heatmap of unsupervised clustering of the CNB and EBS using the Oncotype Dx genes. Unsupervised clustering heatmap of all samples using the Oncotype Dx genes only. 18 of the 21 paired biopsies co-aggregated in the first level dendrogram. Lower heatmap shows the Nanostring expression levels of the control housekeeping genes.

**Figure 4:**
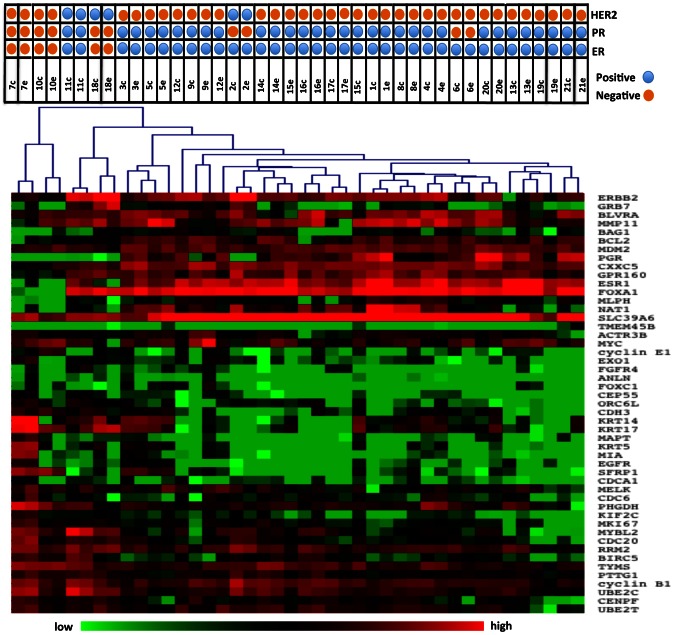
Heatmap of unsupervised clustering of the CNB and EBS using the PAM50 genes. Unsupervised clustering heatmap of all samples using the PAM50 genes only. 19 of the 21 paired biopsies co-aggregated in the first level dendrogram.

The samples were clustered in order to determine whether core and excisional biopsies aggregated together as nearest neighbors in clustering dendrograms. All the paired samples of cores and excisions co-aggregated at the first level dendrogram (figure 2). Additionally, when we clustered the samples using just the 16 Oncotype Dx genes, 18/21 (85%) of the paired samples co-aggregated at the level of the first dendrogram (figure 3). Clustering of the samples by the PAM50 gene set yielded similar results with 19/21 of the paired samples co-aggregating (figure 4). Although, in this study we used the Nanostring for gene expression analysis and not quantitative reverse-transcriptase PCR as done in the Oncotype Dx and PAM50 studies, our results imply that the expression of the genes that comprise the Oncotype Dx score and the risk of relapse (ROR) score may be comparable between a core biopsy and excisional biopsy.

**Figure 5:**
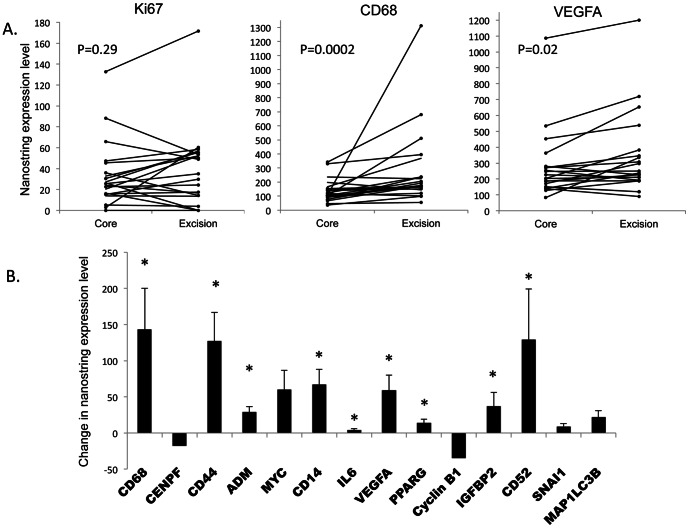
Changes in the expression levels of immune related genes between CNBs and EBs. A. Individual patient plots (n = 21) for NanoString expression level at the time of the CNB and EB for Ki67, CD68, and VEGFA. P-values are from Wilcoxon signed rank tests. B. Bar graph represents the Nanostring expression value differences between the CNB and EBs for all the genes that had a statistically significant change (by Wilcoxon signed rank tests). * denotes genes with immune related functions.

We next analyzed each of the 147 genes individually and used the Wilcoxon's signed rank test to compare the Nanostring expression level between the core and excisional biopsy (complete analysis in table S2). The majority of the genes, including MKI67 (Ki67) did not differ significantly between the core and excisional biopsies without therapeutic intervention. However, there were 14 genes that did significantly differ (p<0.05) as shown in figure 5. Of the 14 genes 9 have functions that are immune related, consisting of: markers of macrophages and monocytes (CD68, CD14), markers of lymphocytes or lymphocyte activation (CD52, CD44), cytokines or other factors that either activate immune cells or are modulated by immune related signals (IL6, PPARG, ADM, IGFBP2, VEGFA) [23]–[27]. The digital expression level of all of these nine immune related transcripts increased in the excisional biopsy compared to the matched core biopsy. The increase in the expression levels of three of the immune related transcripts (CD68, CD52 and CD14) was also validated by real-time PCR (figure S1). The increased expression of immune related genes in the excisions is likely due to an inflammatory response to the core biopsy itself and not because of intra-tumoral heterogeneity. This is supported by the comparison of the scatter plots of the Nanostring gene expression levels obtained from multiple biopsies taken from the same tumor specimen at the same time point but from different sites, which did not reveal any difference in gene expression levels, as shown in additional file, figure S2. However due to limited sample size no hypothesis testing was done to assess the difference in gene expression level between these biopsy samples. Importantly, the increases in expression of CD68 and the other immune related genes were not related to the time interval between the CNB and EB (additional file, table S3).

**Figure 6:**
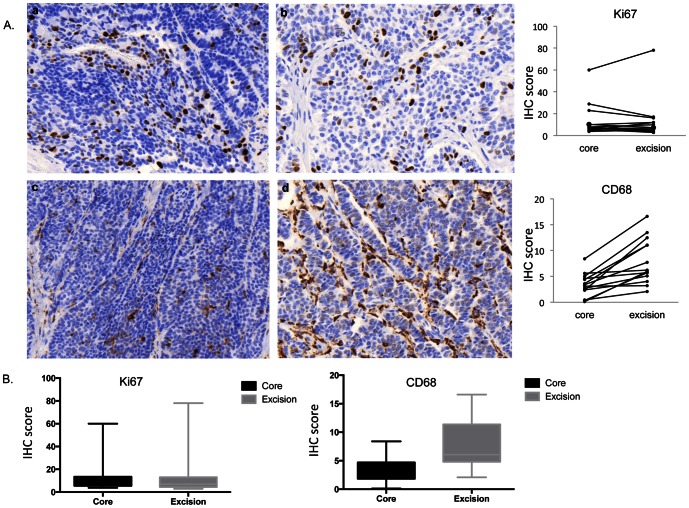
Immunohistochemistry analysis of CNBs and EBs. **a** and **b** represent Ki67 staining of CNB and EB respectively and **c** and **d** represent CD68 staining in a CNB and EB. Magnification 20×. A. Box plots of the Immunohistochemistry scoring for Ki67 and CD68 in the CNB samples and EBs.

In order to corroborate the results of the Nanostring transcript expression level with protein expression we performed immunohistochemistry staining for Ki67 and CD68 in 14 of the CNBs and matched EBs. We chose CD68, because the Nanostring expression level of this gene was most significantly increased in the EBs compared to the CNBs. We detected higher CD68 positive cells in the EBs compared to the CNB (median difference 5.4, IQR 1.6 to 7.4; P = 0.0002) and no significant difference in Ki67 (median difference −1.0, IQR −3.4 to +2.2; P = 0.63), which is consistent with the transcript expression levels (Figure 6). Interestingly, the recruitment of the CD68 positive cells, which are tumor-associated macrophages (TAMs), is seen mainly among the invasive tumor cells and to a lesser extent in the associated stroma.

## Discussion

A CNB followed by a short interval and definitive surgery is the standard practice in the care of early stage breast cancer patients. This time interval provides an opportunity for trials involving a brief exposure to new agents with the goals of validating the ability of the agent to hit its target and for the identification of potential biomarkers that can predict responsiveness to the drug. Several such studies, defined as window of opportunity studies or non-therapeutic studies, have already been completed and have used the Ki67 changes as a surrogate marker for response and as the primary aim. Some of these studies did not include a control arm to validate that the biological changes seen after a brief exposure to a specific agent are indeed due to the exposure and not variations due to sampling differences. The notion that gene expression variations may exist between CNB and EBs is buttressed by the controversial data derived from multiple studies that investigated the concordance of the hormone receptor (HR) status (ER and PR) in CNBs compared to EBs [28]–[30]. A recent meta-analysis of 21 articles showed that overall the agreement between the CNB and EB was high, but there was a meaningful difference in the expression levels of HR markers determined by immunohistochemistry, particularly for PR (7.2% and 14.8% discordance for ER and PR, respectively) [31]. To our knowledge, a large-scale analysis of the variations in gene expression between CNB and EB without any intervention has not been conducted and this was the aim of this study.

We compared CNBs and EBs collected prospectively from 21 patients with newly diagnosed early stage breast cancer. All patients were postmenopausal and the majority had HR positive disease. We studied the quantitative expression levels of 147 breast cancer associated transcripts using the Nanosting nCounter platform and found that for the majority of the transcripts there was high concordance between the CNBs and EBs. The list of transcripts included in this study comprised of genes with known functions in the ER transcriptional program, ER resistance pathways and genes with prognostic significance in HR positive tumors, since these genes have the potential to also have predictive properties for novel treatments in HR positive tumors. MKI67 (Ki67) is a key transcript in this analysis since it has been shown to be a prognostic factor in breast cancer and the change in Ki67 after a short exposure to hormonal therapy has been shown to be predictive of long term outcomes in HR positive tumors though this has not been validated in the other sub-types of breast cancer. In our study we did not find Ki67 variability between the CNB and EB without any treatment at the RNA and protein expression levels and therefore, changes seen in Ki67 levels after a brief exposure to a treatment, are likely due to the exposure and not because of differences in sampling.

Our study is notable for significant differences between CNBs and EBs in the expression level of 14 transcripts of which the majority are immune related. Of particular interest is the difference in CD68, a marker for TAMs. In human breast tumors, infiltrating TAMs correlate with poor prognostic features, higher tumor grade and decreased disease free survival [32], [33]. This may be, in part due to increased angiogenesis that has been shown in mouse models of mammary gland tumors and in human breast tumors where CD68 levels correlate with VEGF expression [34], [35]. In a recent study, blockade of TAMs recruitment in addition to chemotherapy in mammary tumors in MMTV-polyoma middle T antigen mice led to a reduction in primary tumor progression and metastasis and improved survival [36]. Thus, targeting TAMS is a potential new approach for breast cancer treatment and the increase in CD68 and other immune related genes between the CNBs and EBs has several implications; first, window of opportunity clinical trials for immune modulating drugs and in general, changes in immune related genes after exposure to a drug may be difficult to interpret due to the inflammatory changes that are unrelated to drug exposure as shown in this study. Second, although our study is limited as we studied a relatively small number of immune related genes and the immune microenviroment in breast cancer is complex, the recruitment of CD68 positive TAMs after a CNB raises the concern that this recruitment may occur after each breast cancer biopsy. This raises the question of whether CNB may have a deleterious effect on outcome through the stimulation of the recruitment of TAMs and this is of particular concern as many neoadjuvant clinical trials include additional biopsies. Hence, studies of the immune effects of multiple biopsies in early stage disease seem warranted.

## Conclusions

In summary, although this study has limitations because of the number of patients and number of transcripts evaluated, we show that overall there are not many gene expression variations between CNBs and EBs and therefore the differences in the sampling methods between these two types of biopsies is not a significant drawback for window of opportunity studies. However, even in this small study we were able to detect significant differences in the expression of immune related genes that will need to be considered when interpreting window of opportunity studies. We also found a strong correlation between the clinical data, based on immunohistochemistry staining and established guidelines and the Nanostring nCounter expression value, suggesting that this platform could be a useful tool in clinical studies that incorporate gene expression analysis.

## Supporting Information

Figure S1 **Increase in expression of immune related genes in the EB validated by RT-PCR.** The fold change between CNB and EB of the expression of immune related genes (CD68, CD52, CD14) compared to non-immune related genes (ESR1, ERBB2, MKI67, PTEN) was significantly higher in the immune related genes (p<0.001, Student's t-test).
(TIF)

Figure S2 **Multiple same time biopsies in individual patients.** Logged expression values of the 14 genes that were significantly different between the CNBs and EBs in biopsies obtained from different sites at the same time from 3 individual patients.
(TIFF)

Table S1 **Nanostring gene expression analysis.** Normalized nanostring gene expression values for the 147 transcripts.
(DOCX)

Table S2 **Gene expression differences between CNB and EB.** The table includes the mean, median and standard of change between the biopsies and the p-values for the Wilcoxon's signed rank test.
(XLSX)

Table S3 **The increase in immune responsive gene expression at the time of the EB is not dependent on the time interval between the CNB and EB.** Wilcoxon's rank sum tests were performed to evaluate the association of the difference in gene expression between the two biopsies and the time interval between the biopsies. The time interval is dichotomized at 1 month.
(DOCX)

Table S4 **Oligonucleotide primer sequences used for RT-PCR.**
(DOCX)
