# Supplementary figures and images for: Digital Quantification of Gene Expression in Sequential Breast Cancer Biopsies Reveals Activation of an Immune Response
Source: PLoS One. 2013 May 31;8(5):e64225. doi: 10.1371/journal.pone.0064225 (PMC3669373; doi:10.1371/journal.pone.0064225)

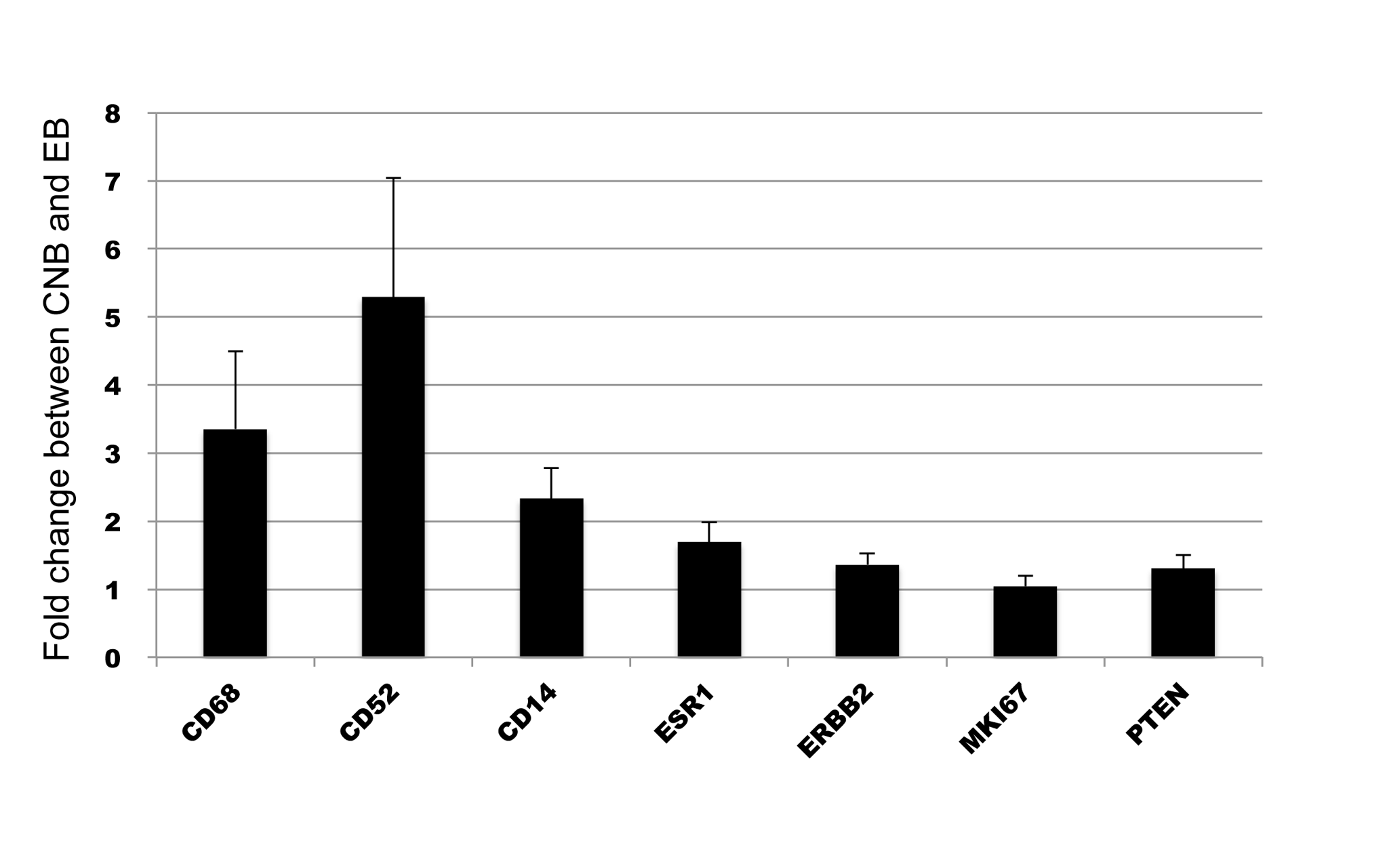

Supplement: Figure S1 — Increase in expression of immune related genes in the EB validated by RT-PCR. The fold change between CNB and EB of the expression of immune related genes (CD68, CD52, CD14) compared to non-immune related genes (ESR1, ERBB2, MKI67, PTEN) was significantly higher in the immune related genes (p<0.001, Student's t-test). (TIF) [file pone.0064225.s001.tif]

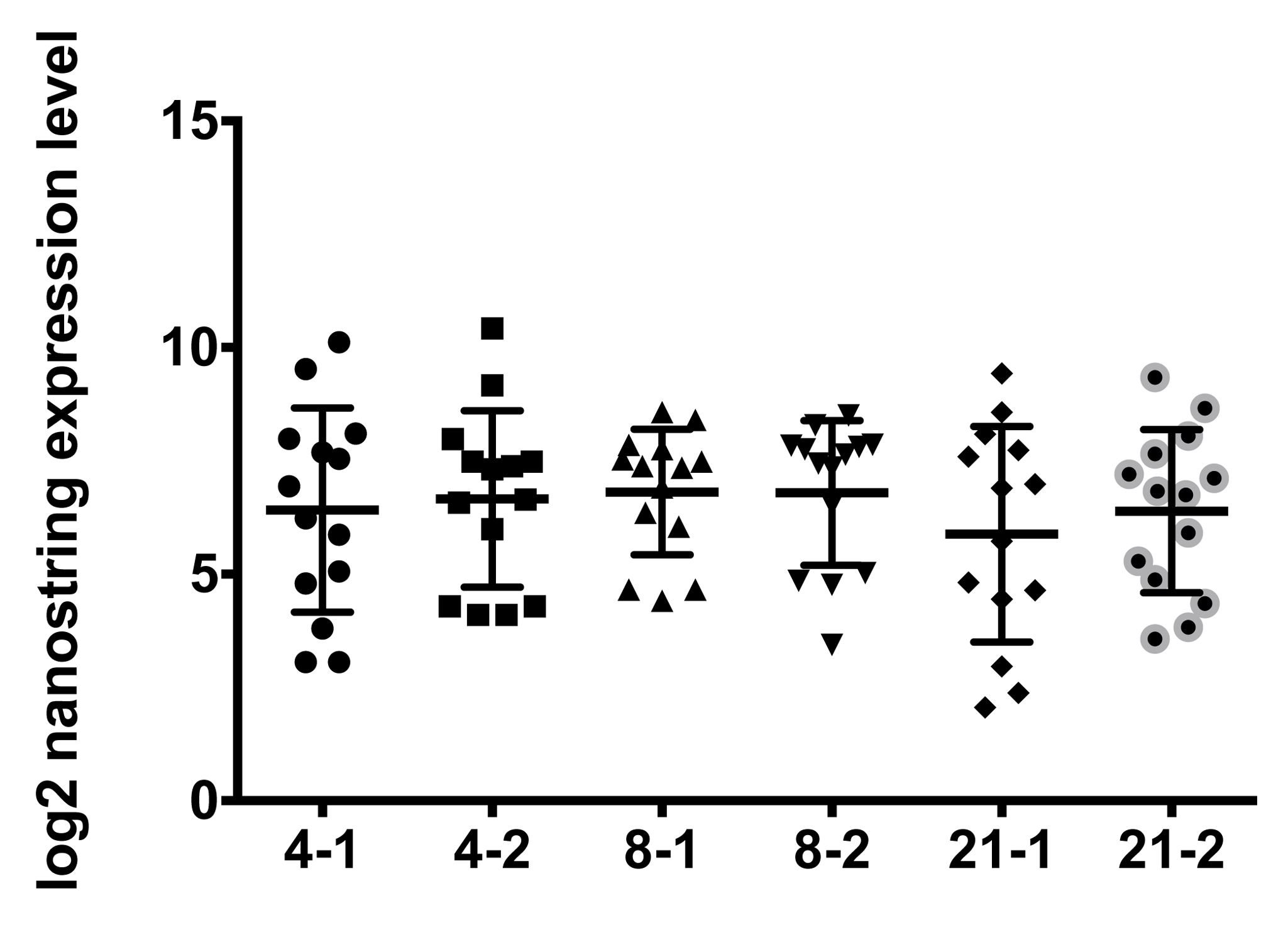

Supplement: Figure S2 — Multiple same time biopsies in individual patients. Logged expression values of the 14 genes that were significantly different between the CNBs and EBs in biopsies obtained from different sites at the same time from 3 individual patients. (TIFF) [file pone.0064225.s002.tiff]
