# Supplementary material for: Digital Quantification of Gene Expression in Sequential Breast Cancer Biopsies Reveals Activation of an Immune Response
Source: PLoS One. 2013 May 31;8(5):e64225. doi: 10.1371/journal.pone.0064225 (PMC3669373; doi:10.1371/journal.pone.0064225)
Supplement: Table S1 — Nanostring gene expression analysis. Normalized nanostring gene expression values for the 147 transcripts. (DOCX) [file pone.0064225.s003.docx]

Table S1. Oligonucleotide primer sequences used for RT-PCR

| **Target gene** | **Primer designation** | **Sequence (5 ^/^-3^/^)** |
| --- | --- | --- |
| CD68 | 5^/^-CD68 | CCACCTGCTTCTCTCATTCC |
|  | 3^/^-CD68 | TTGTACTCCACCGCCATGTA |
| CD52 | 5^/^-CD52 | GCCACGAAGATCCTACCAAA |
|  | 3^/^-CD52 | GCTGAGACGTGTCACCTCAA |
| CD14 | 5^/^-CD14 | GCCGCTGTGTAGGAAAGAAG |
|  | 3^/^-CD14 | GCTGAGGTTCGGAGAAGTTG |
| ESR1 | 5^/^-ESR1 | CAAGCCCATGGAACATTTCT |
|  | 3^/^-ESR1 | TCCCTTGGATCTGATGCAGT |
| ERBB2 | 5^/^-ERBB2 | CTCAGCGTCTTCCAGAACCT |
|  | 3^/^-ERBB2 | AGCAGAGGTGGGTGTTATGG |
| MKI67 | 5^/^-MKI67 | AAATTTGCTTCTGGCCTTCC |
|  | 3^/^-MKI67 | GGAAGCTGGATACGGATGTC |
| PTEN | 5^/^-PTEN | AGCAGCTTCTGCCATCTCTC |
|  | 3^/^-PTEN | TCTGCAGGAAATCCCATAGC |
