# Supplementary material for: Digital Quantification of Gene Expression in Sequential Breast Cancer Biopsies Reveals Activation of an Immune Response
Source: PLoS One. 2013 May 31;8(5):e64225. doi: 10.1371/journal.pone.0064225 (PMC3669373; doi:10.1371/journal.pone.0064225)
Supplement: Table S3 — The increase in immune responsive gene expression at the time of the EB is not dependent on the time interval between the CNB and EB. Wilcoxon's rank sum tests were performed to evaluate the association of the difference in gene expression between the two biopsies and the time interval between the biopsies. The time interval is dichotomized at 1 month. (DOCX) [file pone.0064225.s005.docx]

Table S3. The gene expression changes between the CNB and EB.

| gene | Change (mean) | Std (mean) | Med (change) | Signed rank test stats | Signed rank test p-value |
| --- | --- | --- | --- | --- | --- |
| CD68_MOLECULE | 142.67 | 269.05 | 74.13 | 95.5 | 0.000138 |
| CENPF | -17.81 | 20.72 | -18.51 | -87 | 0.000483 |
| CD44 | 126.64 | 191.15 | 70.88 | 87.5 | 0.000759 |
| ADM | 28.85 | 35.90 | 9.60 | 74 | 0.001694 |
| MYC | 60.20 | 126.09 | 42.52 | 80.5 | 0.002544 |
| CD14 | 66.56 | 100.53 | 58.42 | 79.5 | 0.002973 |
| IL6 | 4.33 | 9.42 | 0.00 | 17 | 0.015625 |
| VEGFA | 59.30 | 100.31 | 39.37 | 66.5 | 0.016676 |
| PPARG | 13.75 | 23.50 | 3.17 | 44 | 0.021393 |
| CYCLIN_B1 | -35.23 | 76.28 | -16.71 | -62.5 | 0.025825 |
| IGFBP2 | 36.93 | 88.51 | 20.99 | 62.5 | 0.025825 |
| CD52 | 129.31 | 327.69 | 52.90 | 61.5 | 0.028645 |
| SNAI1 | 9.18 | 18.59 | 3.51 | 48.5 | 0.034233 |
| MAP1LC3B | 21.83 | 44.50 | 8.42 | 56.5 | 0.046603 |
| EXO1 | 7.52 | 17.30 | 4.61 | 48 | 0.054573 |
| CARM1 | 16.17 | 42.31 | 16.90 | 54.5 | 0.055847 |
| MUC1 | -113.23 | 279.36 | -99.43 | -54.5 | 0.055847 |
| CDKN1A | 19.46 | 45.08 | 20.76 | 53.5 | 0.060967 |
| FOSL1 | 17.58 | 39.31 | 1.36 | 39.5 | 0.063828 |
| TNFRSF21 | 11.39 | 24.50 | 15.85 | 52.5 | 0.066438 |
| CD28 | 3.48 | 7.59 | 0.00 | 21 | 0.067383 |
| C21ORF34 | 8.16 | 18.01 | 0.00 | 29.5 | 0.067627 |
| FOXO3A | 27.52 | 75.52 | 36.11 | 51.5 | 0.072275 |
| VIM | 828.19 | 2197.74 | 787.69 | 50.5 | 0.07849 |
| SPDEF__PDEF_ | -45.05 | 106.95 | -42.64 | -47 | 0.08255 |
| CD24 | -212.70 | 767.21 | -122.43 | -47.5 | 0.099547 |
| BAG1 | -4.02 | 14.46 | -3.21 | -36.5 | 0.118706 |
| PAX2 | 4.03 | 10.56 | 0.00 | 18 | 0.123047 |
| MLPH | -8.93 | 21.98 | -11.66 | -42 | 0.123093 |
| MMP2 | 81.86 | 385.14 | 115.95 | 44.5 | 0.124492 |
| VAV1 | 3.86 | 11.74 | 2.32 | 34.5 | 0.141518 |
| MMP11 | -44.85 | 178.41 | -62.07 | -41.5 | 0.153645 |
| HSD17B2 | 3.23 | 8.08 | 0.00 | 7.5 | 0.15625 |
| DST | 10.06 | 45.63 | 10.79 | 40.5 | 0.164346 |
| PHGDH | -23.89 | 60.99 | -15.58 | -39.5 | 0.175555 |
| IL6R | 6.05 | 19.73 | 5.24 | 37 | 0.176853 |
| TNFSF8 | 8.58 | 19.77 | 0.00 | 24 | 0.187622 |
| CCND1 | -47.19 | 342.81 | -72.95 | -37.5 | 0.199525 |
| MELK | 25.10 | 67.79 | 21.59 | 37.5 | 0.199525 |
| MAPK1 | 7.45 | 35.37 | 10.79 | 36.5 | 0.2123 |
| AR | -36.24 | 137.65 | -28.15 | -34 | 0.216167 |
| CLDN7 | -34.61 | 123.73 | -16.86 | -35.5 | 0.225608 |
| KRAS | 10.07 | 59.13 | 17.38 | 35.5 | 0.225608 |
| FOXM1 | 8.22 | 27.97 | 5.89 | 34.5 | 0.239455 |
| UBE2C | -28.49 | 96.03 | -10.27 | -34.5 | 0.239455 |
| AURKB | -3.51 | 11.64 | -0.51 | -25.5 | 0.2435 |
| CTSL2 | 2.52 | 7.46 | 0.00 | 3 | 0.25 |
| KRT18 | -113.51 | 456.62 | -201.80 | -33.5 | 0.253842 |
| TP53 | -8.83 | 34.47 | -15.27 | -33.5 | 0.253842 |
| HER3 | -61.61 | 189.00 | -35.32 | -32.5 | 0.268773 |
| ORC6L | 4.77 | 17.83 | 0.00 | 22 | 0.274445 |
| BIRC5_SURVIVIN | -3.61 | 21.02 | -5.56 | -30 | 0.277355 |
| KRT17 | 11.75 | 75.20 | 3.28 | 23.5 | 0.284164 |
| RRM2 | -17.00 | 61.33 | -1.13 | -31.5 | 0.284249 |
| SCUBE2 | -10.70 | 364.86 | -31.90 | -31.5 | 0.284249 |
| MKI67 | 5.70 | 22.85 | 1.66 | 29 | 0.294252 |
| ACTR3B | -5.79 | 23.23 | -4.62 | -29.5 | 0.316834 |
| FGFR4 | 3.30 | 10.94 | 0.00 | 12 | 0.320313 |
| ALPHA6_INTEGRIN_CD49F | 7.72 | 38.48 | 13.26 | 28.5 | 0.333939 |
| CYCLIN_E2 | -7.00 | 26.49 | -5.09 | -28.5 | 0.333939 |
| FOXC1 | 5.66 | 19.70 | 0.00 | 13 | 0.339355 |
| BRCA1 | 6.60 | 18.98 | 0.00 | 22.5 | 0.34655 |
| ZWINT | 1.31 | 33.06 | -10.89 | -25.5 | 0.388462 |
| CDC20 | 9.48 | 31.68 | 0.73 | 23 | 0.409098 |
| CHEK1 | 0.48 | 11.33 | 4.30 | 23.5 | 0.427421 |
| PDK1 | 6.00 | 23.05 | 0.64 | 23.5 | 0.427421 |
| CDH3 | -4.98 | 28.72 | -1.46 | -17.5 | 0.430679 |
| TMEM45B | 3.05 | 13.58 | 0.00 | 3.5 | 0.4375 |
| ESR1 | 19.84 | 229.93 | 14.98 | 21 | 0.452375 |
| MIA | -2.30 | 14.28 | 0.00 | -11.5 | 0.454834 |
| ANLN | 2.58 | 8.88 | 0.00 | 6 | 0.460938 |
| AURORA_KINASE_A_STK15 | 1.69 | 7.61 | 0.00 | 6 | 0.460938 |
| GATA3 | 9.08 | 159.02 | 11.64 | 21.5 | 0.468389 |
| GRHL2 | -2.65 | 28.67 | -8.37 | -21.5 | 0.468389 |
| CD79B | 2.30 | 11.09 | 0.00 | 10 | 0.469727 |
| AIB1 | 5.69 | 33.43 | 8.14 | 20.5 | 0.489597 |
| MYBL1 | 4.73 | 18.70 | 0.00 | 16.5 | 0.495079 |
| RAD54B | 1.59 | 13.11 | 0.00 | 14 | 0.495422 |
| LXN | 6.74 | 60.08 | 3.86 | 19 | 0.498009 |
| NAT1 | -19.59 | 151.46 | -9.91 | -19 | 0.498009 |
| CYP24A1 | 0.23 | 0.74 | 0.00 | 2 | 0.5 |
| ESR2 | 0.43 | 1.47 | 0.00 | 1.5 | 0.5 |
| PABPC1 | 7.70 | 173.20 | 62.00 | 19.5 | 0.511273 |
| GRB7 | 19.39 | 88.27 | 0.00 | 15.5 | 0.522614 |
| CLDN4 | 18.12 | 176.70 | 33.12 | 18.5 | 0.5334 |
| EVL | 14.58 | 100.03 | 14.26 | 18.5 | 0.5334 |
| SNAI2 | 7.44 | 56.53 | 15.98 | 18.5 | 0.5334 |
| INSULIN_RECEPTOR | 14.58 | 89.32 | 1.16 | 17.5 | 0.555964 |
| MDM2 | 7.92 | 61.67 | 11.02 | 17.5 | 0.555964 |
| PLEKHF2 | -66.91 | 223.78 | 3.15 | -17.5 | 0.555964 |
| SLC7A5 | 8.81 | 54.56 | 10.49 | 17.5 | 0.555964 |
| CALCR | 1.93 | 9.16 | 0.00 | 4 | 0.578125 |
| TGFB3 | 0.92 | 54.73 | 6.25 | 16.5 | 0.578948 |
| TFF3 | 223.55 | 1141.36 | 8.51 | 15 | 0.595819 |
| FOXA1 | -49.51 | 396.59 | -47.19 | -14 | 0.621513 |
| CYP27B1 | 1.12 | 14.44 | 0.00 | -2.5 | 0.625 |
| EGFR | 1.15 | 9.45 | 0.00 | 8.5 | 0.625732 |
| PI3K | 3.11 | 34.96 | 3.42 | 14.5 | 0.626104 |
| PTPN12 | -0.16 | 25.59 | 5.14 | 13.5 | 0.650238 |
| BRCA2 | -1.76 | 23.08 | 1.88 | 12 | 0.674223 |
| ESRP1 | -12.22 | 72.97 | -12.01 | -12.5 | 0.674716 |
| KIF2C | 1.15 | 16.02 | 0.00 | 9.5 | 0.677704 |
| NDC80__KNTC2 | -0.38 | 14.18 | -0.04 | -11 | 0.679428 |
| P27 | 3.87 | 47.11 | 5.10 | 11.5 | 0.699515 |
| GSTM1 | -33.72 | 253.01 | 0.00 | -5 | 0.700195 |
| TRPV6 | -5.79 | 29.93 | 0.00 | -5 | 0.700195 |
| CYCLIN_E1 | 2.04 | 14.67 | 0.00 | 9.5 | 0.701881 |
| KIT | -1.37 | 17.18 | 0.00 | -8.5 | 0.711899 |
| CXXC5 | -5.83 | 63.40 | -8.72 | -9.5 | 0.749988 |
| IRS1 | -9.89 | 54.13 | -2.88 | -9.5 | 0.749988 |
| XBP1 | -115.86 | 1172.03 | 40.15 | 9.5 | 0.749988 |
| CAMP | 0.72 | 4.99 | 0.00 | 1 | 0.75 |
| PGR | 19.49 | 228.03 | 0.00 | 7.5 | 0.766029 |
| AKT | -6.61 | 166.51 | -9.94 | -8.5 | 0.775615 |
| BLVRA | 2.71 | 114.48 | -30.45 | -8.5 | 0.775615 |
| IL6ST | 19.69 | 545.56 | 11.74 | 8.5 | 0.775615 |
| KRT14 | 26.66 | 232.31 | 0.00 | 6.5 | 0.781906 |
| ELF5 | -10.53 | 59.24 | 0.00 | 4 | 0.791016 |
| PTTG1 | -1.32 | 34.56 | 0.01 | -7.5 | 0.80147 |
| MAPT | 1.81 | 16.19 | 0.00 | -4.5 | 0.807739 |
| MYBL2 | -15.82 | 72.90 | 0.00 | -7 | 0.812355 |
| LIF | 0.62 | 6.08 | 0.00 | 1.5 | 0.84375 |
| GPR126 | -7.15 | 38.93 | 0.00 | -4 | 0.846924 |
| BCL2 | 3.13 | 65.07 | 6.94 | 5.5 | 0.853759 |
| ERBB2 | 44.75 | 316.77 | 6.74 | -5.5 | 0.853759 |
| POLR2K | -21.47 | 159.05 | -7.33 | -5.5 | 0.853759 |
| SFRP1 | -14.45 | 60.95 | 0.00 | 4 | 0.86026 |
| TSPYL5 | -1.51 | 45.13 | 0.25 | -5 | 0.869488 |
| ATP6V1C1 | 0.52 | 24.74 | -1.65 | 4.5 | 0.880142 |
| TIMP3 | -118.72 | 837.18 | -14.04 | -4.5 | 0.880142 |
| CDC6 | 7.66 | 36.99 | -2.65 | -4 | 0.898317 |
| GPR160 | -13.35 | 134.43 | 13.02 | 3.5 | 0.906649 |
| MAP1LC3A | 0.92 | 44.01 | 0.07 | 3.5 | 0.906649 |
| UBE2T | 0.23 | 32.90 | -2.65 | -3.5 | 0.906649 |
| CDH1 | -62.86 | 636.51 | 13.86 | -2.5 | 0.933252 |
| INTS8 | 0.01 | 36.73 | -5.08 | 2.5 | 0.933252 |
| RPL30 | -201.31 | 1948.93 | 292.01 | 2.5 | 0.933252 |
| SLC39A6 | 305.33 | 1960.28 | -11.21 | -2.5 | 0.933252 |
| SRC1 | 1.13 | 33.94 | -1.09 | -2.5 | 0.933252 |
| VDR | 1.53 | 32.58 | 5.96 | 2.5 | 0.933252 |
| YWHAZ | 117.39 | 782.75 | -2.74 | -2.5 | 0.933252 |
| MTDH | 0.29 | 109.88 | 4.41 | 1.5 | 0.959923 |
| CDCA1 | 0.47 | 10.31 | 0.00 | 1 | 0.977966 |
| LAPTM4B | 36.99 | 216.02 | -8.83 | -0.5 | 0.986636 |
| PTEN | -6.87 | 80.41 | 18.26 | 0.5 | 0.986636 |
| TYMS | -0.83 | 44.65 | 4.56 | -0.5 | 0.986636 |
| CEP55 | 0.63 | 9.83 | 0.00 | -0.5 | 1 |
| KRT5 | -1.55 | 26.47 | 0.00 | 0.5 | 1 |

The table includes the mean, median and standard of nanostring gene expression change between the biopsies and the p-values for the Wilcoxon’s signed rank test.
