# Supplementary material for: Digital Quantification of Gene Expression in Sequential Breast Cancer Biopsies Reveals Activation of an Immune Response
Source: PLoS One. 2013 May 31;8(5):e64225. doi: 10.1371/journal.pone.0064225 (PMC3669373; doi:10.1371/journal.pone.0064225)
Supplement: Table S4 — Oligonucleotide primer sequences used for RT-PCR. (DOCX) [file pone.0064225.s006.docx]

Table S4. The association between the length of the time interval between the biopsies and gene expression changes

| **Gene** | **Interval** | **N** | **Median** | **Q1** | **Q3** | **Min** | **Max** | **P-value** |
| --- | --- | --- | --- | --- | --- | --- | --- | --- |
| CD68 | <1 mo | 10 | 47.71 | 9.07 | 202.86 | -51.69 | 407.77 | 0.9439 |
| CD68 | ≥1 mo | 11 | 74.13 | 19.48 | 111.52 | -17.51 | 1207.24 | 0.9439 |
| CENPF | <1 mo | 10 | -21.03 | -33.21 | -8.57 | -42.12 | 0.00 | 0.2599 |
| CENPF | ≥1 mo | 11 | -11.22 | -23.31 | -7.56 | -72.88 | 28.82 | 0.2599 |
| CD44 | <1 mo | 10 | 23.74 | 5.14 | 237.27 | -137.21 | 662.05 | 0.1809 |
| CD44 | ≥1 mo | 11 | 96.48 | 65.00 | 220.74 | -4.50 | 539.54 | 0.1809 |
| ADM | <1 mo | 10 | 4.15 | -1.23 | 55.06 | -15.18 | 110.79 | 0.4179 |
| ADM | ≥1 mo | 11 | 31.72 | 1.66 | 55.07 | -5.23 | 92.15 | 0.4179 |
| MYC | <1 mo | 10 | 43.51 | 6.87 | 69.79 | -39.38 | 90.34 | 0.6727 |
| MYC | ≥1 mo | 11 | 42.52 | 16.17 | 107.61 | -106.83 | 554.33 | 0.6727 |
| CD14 | <1 mo | 10 | 53.55 | 5.19 | 125.84 | -85.63 | 194.32 | 0.6727 |
| CD14 | ≥1 mo | 11 | 61.65 | 31.97 | 117.48 | -116.26 | 328.81 | 0.6727 |
| IL6 | <1 mo | 10 | 0.00 | 0.00 | 2.70 | -2.54 | 36.15 | 0.6870 |
| IL6 | ≥1 mo | 11 | 0.00 | 0.00 | 6.41 | 0.00 | 13.26 | 0.6870 |
| VEGFA | <1 mo | 10 | 19.32 | -31.38 | 41.56 | -62.46 | 290.94 | 0.1809 |
| VEGFA | ≥1 mo | 11 | 73.39 | 1.52 | 178.63 | -49.16 | 251.62 | 0.1809 |
| PPARG | <1 mo | 10 | 2.62 | -5.67 | 20.15 | -11.03 | 73.13 | 0.6198 |
| PPARG | ≥1 mo | 11 | 3.17 | 0.00 | 43.94 | -3.58 | 60.78 | 0.6198 |
| Cyclin B1 | <1 mo | 10 | -35.42 | -96.44 | -0.27 | -299.29 | 51.95 | 0.3242 |
| Cyclin B1 | ≥1 mo | 11 | -10.58 | -56.65 | 20.38 | -79.67 | 47.51 | 0.3242 |
| IGFBP2 | <1 mo | 10 | 19.83 | 8.87 | 49.24 | -105.45 | 128.40 | 0.8327 |
| IGFBP2 | ≥1 mo | 11 | 20.99 | -2.87 | 81.92 | -36.77 | 304.92 | 0.8327 |
| CD52 | <1 mo | 10 | 60.15 | -33.65 | 123.53 | -298.11 | 407.86 | 0.5262 |
| CD52 | ≥1 mo | 11 | 52.90 | 16.29 | 212.76 | -150.69 | 1389.6 | 0.5262 |
| SNAI1 | <1 mo | 10 | 5.81 | -64.12 | 44.34 | -123.17 | 124.72 | 0.7248 |
| SNAI1 | ≥1 mo | 11 | 18.48 | -17.54 | 53.46 | -45.67 | 61.57 | 0.7248 |
| MAP1LC3B | <1 mo | 10 | 5.82 | -28.17 | 37.27 | -38.23 | 54.82 | 0.1590 |
| MAP1LC3B | ≥1 mo | 11 | 58.36 | -19.27 | 70.46 | -26.77 | 114.23 | 0.1590 |

**The increase in immune responsive gene expression at the time of the EB is not dependent on the time interval between the CNB and EB** Wilcoxon’s rank sum tests were performed to evaluate the association of the difference in gene expression between the two biopsies and the time interval between the biopsies. The time interval is dichotomized at 1 month.
